# Antithrombin Deficiency Is Associated with a Novel Homozygous Detrimental Mutation in *SERPINC1* Gene in a Saudi Female

**DOI:** 10.1155/2023/8872346

**Published:** 2023-04-20

**Authors:** Sana Alqarni, Baraah Alqarni, Abdulrahman Alsultan

**Affiliations:** ^1^Department of Clinical Laboratory Science, College of Applied Medical Science, King Saud University, Riyadh, Saudi Arabia; ^2^Department of Pediatrics, College of Medicine, King Saud University, Riyadh, Saudi Arabia; ^3^Oncology Center, King Saud University Medical City, Riyadh, Saudi Arabia

## Abstract

Antithrombin (AT) deficiency is a blood disorder associated with an increased tendency to form thrombosis. Hereditary AT deficiency is frequently caused by mutations in *SERPINC1* gene. It is usually inherited as an autosomal dominant with variable penetrance. Homozygous pathogenic mutations in this gene are extremely rare. We present a case of a 7-year-old female who presented at age of 4 years with massive cerebral sinus venous thrombosis. Thrombophilia workup showed a low AT level of 30%. Targeted genetic sequencing of *SERPINC1* revealed a novel pathogenic homozygous mutation c.1320C>G p. (Phe440Leu). The patient was managed initially with unfractionated heparin with AT replacement using fresh frozen plasma and was later switched to only low-molecular-weight heparin. There was no recurrence or new thrombosis with 3 years of follow-up.

## 1. Introduction

**Figure 1:**
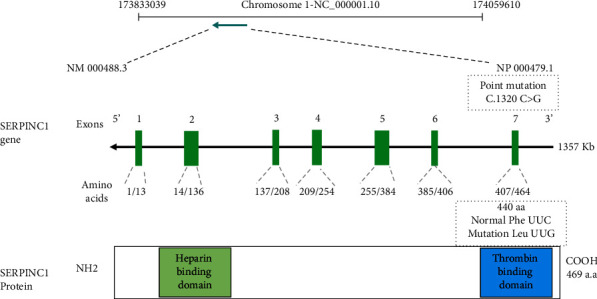
The *SERPINC1* gene and protein domain structures. The figure showed gene ID 462 *SERPINC1* as well as merged features of NP_000479.1 and NM_000488.3 with 1357 kb. The *SERPINC1* gene shown by a heavy horizontal arrow line represents genomic DNA. Exons are shown as vertical green bars and numbered from 1 to 7. The amino acids encoded by each exon are indicated below the vertical green bars and are numbered from 1 to 464. The *SERPINC1* protein structure showed two main domains: the NH_2_-terminal heparin-binding domain encoded by exon 2 and a COOH-terminal thrombin- (serine protease-) binding domain. The patient point mutation is indicated by red colour in the dotted box at exon 7 (c.1320C>G); also, the result of this mutation is indicated on 440 a.a which leads to produced Leu (leucine) instead of Phe (phenylalanine) (adapted from [9, 10]).

Antithrombin (antithrombin III, AT) deficiency is a thrombophilia disorder. AT is a significant plasmatic inhibitor for activated coagulation factors such as thrombin (factor IIa), factor Xa, and factor IXa [1]. AT protein is encoded by the *SERPINC1* (serpin family C member 1) gene. *SERPINC1* is located on chromosome 1q25.1 and contains seven exons and six introns. The structure of *SERPINC1* gene and protein is shown in Figure 1. Hereditary AT deficiency is usually caused by single-point mutations in *SERPINC1*. It is associated with an about 20-fold increase in the incidence of venous thromboembolism (VTE) [2]. Several mutations and genetic alterations have been recognized in this gene which is relatively rare (1 in 2000–3000 individuals) [3, 4]. According to ClinVar on the NIH website [5], there are 300 different SERPINC1 mutations causing AT deficiency. Most of the mutations that have been described in the *SERPINC1* are heterozygous, which are inherited as autosomal dominant [6]. Homozygosity is extremely rare and may not be compatible with life [3]. However, a few patients who harbour homozygous mutations were reported and had severe thromboembolic events early in life [7, 8]. In most AT deficiency, patients showed a decrease in AT plasmatic level to less than 50% (normal range, 80–120%).

There are two types of AT deficiency: quantitative (type I) and qualitative (type II). Quantitative (type I) AT deficiency occurs when the AT level is reduced, and the antigen/anticoagulant activity ratio is close to one. Patients with qualitative (type II) AT deficiency have normal antigen levels; however, the antigen/anticoagulant activity ratio is higher than one, indicating that a variant AT with impaired or null anticoagulant activity is present in plasma. Type II AT deficiency can be classified into three subtypes according to the AT functional defect, which is frequently associated with the location of the pathogenic mutation: IIa in the reactive site (RS), IIb in the heparin-binding site (HBS), and IIc in pleiotropic (PE) [3].

In this case, we report a 7-year-old child with a novel homozygous detrimental mutation in *SERPINC1* who presented at the age of 4 years with severe cerebral sinus venous thrombosis.

## 2. Case Presentation

We present the case of a 7-year-old female who presented at the age of 4 years with a history of fever, frequent vomiting, headache, and neck pain. The past medical history is remarkable for two episodes of simple febrile seizures at 2 and 3 years of age. The initial physical assessment showed that the patient was drowsy, had stiff neck, had a Glasgow coma scale of 12/15, and had a normal otherwise neurological assessment. The initial impression was of meningoencephalitis, which was managed with broad-spectrum antibiotics. The septic workup was negative. Brain magnetic resonance imaging (MRI), MR angiography (MRA), and MR venography (MRV) showed an extensive dural sinus and cerebral venous thrombosis with secondary sequela of bilateral thalamic and basal ganglia venous congestion and venous infarcts. The patient was started on unfractionated heparin. Antiphospholipid antibody screening was negative. Initial thrombophilia workup was remarkable for AT level of 14% (normal range, 80–120%). Thus, fresh frozen plasma (FFP) infusion was added to anticoagulation to replace AT. The patient was subsequently switched to enoxaparin with regular FFP infusions in the first year of therapy. The FFP was discontinued, and the patient was continued on enoxaparin with an anti-Xa level ranging between 0.2 and 0.4 IU/mL over the last two years, with no evidence of new or recurrent thrombosis. Oral anticoagulant was not given based on parental preference. AT level ranges now between 25 and 35%.

*SERPINC1* gene sequencing targeted the following: the coding regions, 10 bp of flanking introns, and any previously described pathogenic/likely pathogenic variants. This also included copy number variation analysis. DNA sequencing of *SERPINC1* gene revealed a novel homozygous variant NM_000488.3: c.1320C>G p. (Phe440Leu), which was not reported in Genome Aggregation Database (gnomAD), Exome Sequencing Project (ESP), and 1000 Genomes Project (1000G). AT level was 56% in the father and 50% in the mother, and the older sister had AT level of 61%. The mother had a history of deep vein thrombosis associated with the use of oral contraceptives.

## 3. Discussion

Homozygosity of *SERPINC1* mutation in our patient was associated with type I AT deficiency. The patient presented with thrombosis at the age of 4 years, which was triggered by infection and dehydration. Most of the previously reported autosomal recessive AT deficiency cases developed severe arterial or venous thrombosis early in life [11, 12].

The prevalence of inherited bleeding disorders varies from one population to another due to genetic differences and ethnicity. Little is known about bleeding disorder prevalence in the Saudi population. However, a study by Al Saleh et al. showed that the Saudi population has a higher prevalence of coagulation factor deficiency than the western population [13]. Fewer data are available about AT deficiency in children due to the rarity of the disorder. Thus, the purpose of this report is to increase physician awareness about this rare severe illness.

The molecular genetic basis of AT deficiency has been extensively examined. AT deficiency is caused by 300 distinct *SERPINC1* mutations according to ClinVar NIH website [5]. To minimize this number, we only choose the pathogenic clinically significant and single nucleotide variant type. This gave us 34 clinical variants out of the 300 variants shown in Table 1 in the supplementary. The variant we presented in this article was not reported before. To understand this variant effect in our patient, we locate *SERPINC1* point mutation on the gene structure, and c.1320C>G is in exon 7 in the C-terminal region of *SERPINC1* protein, as shown in Figure 1. The *SERPINC1* protein structure showed two main domains: the NH_2_-terminal heparin-binding domain encoded by exon 2 and a COOH-terminal thrombin- (serine protease-) binding domain encoded by exon 7. Exon 7 is transcribed to mRNA and then translated to amino acid numbers from 407 to 464, which contain the thrombin-binding domain. As the point mutation in our patient is located in exon 7, this mutation may affect the thrombin-binding activity on *SERPINC1* protein which led to thrombosis in this case.

There are no clear guidelines on the optimal duration of anticoagulation in patients with homozygous AT deficiency. Long-term anticoagulation was continued in our patient because of concerns about the high risk of the development of new thrombosis which has been reported in cases before [14]. The optimal choice of anticoagulants (low-molecular-weight heparin, vitamin K antagonists, or direct oral anticoagulant) in AT deficiency still needs to be defined [15].

## 4. Conclusions

Homozygous in *SERPINC1* gene is a rare condition that could cause severe thrombosis. Therefore, patients with homozygous AT deficiency should continue using anticoagulation for life due to the increased risk of thrombus recurrence assumed from previous cases [14]. However, the optimal choice of anticoagulants such as using low-molecular-weight heparin, vitamin K antagonists, or direct oral anticoagulants in AT deficiency still needs to be defined. This report's objective is to make physicians more aware of this severe and uncommon sickness. Furthermore, more investigation about the effect of this mutation on the patients and the way to overcome its consequence is required.

## Data Availability

The DNA sequencing data and laboratory tests used to support the findings of this case or any other data related to this case are available from the corresponding author on reasonable request.

## Supplementary Materials

Supplementary Materials Additional file has Table that provides a review of all previous investigations involving different single-point mutations in the *SERPINC1* gene and clinical manifestations according to ClinVar on the NIH website [5].
