# Supplementary material for: Antithrombin Deficiency Is Associated with a Novel Homozygous Detrimental Mutation in SERPINC1 Gene in a Saudi Female
Source: Case Rep Med. 2023 Apr 20;2023:8872346. doi: 10.1155/2023/8872346 (PMC10139800; doi:10.1155/2023/8872346)
Supplement: Supplementary Materials — Additional file has Table that provides a review of all previous investigations involving different single-point mutations in the SERPINC1 gene and clinical manifestations according to ClinVar on the NIH website [5]. [file 8872346.f1.docx]

### Supplementary

Table 1: provides an overview of all previous investigations involving different single point mutations in the SERPINC1 gene and clinical manifestations according to ClinVar on NIH website (5).

| Genetic Variation | Condition(s) | Clinical significance (Last reviewed) |
| --- | --- | --- |
| [c.1382C>T (p.Pro461Leu)](https://www.ncbi.nlm.nih.gov/clinvar/variation/18031/) | Hereditary antithrombin deficiency | Pathogenic(Sep 6, 2017) |
| [c.1316C>T (p.Pro439Leu)](https://www.ncbi.nlm.nih.gov/clinvar/variation/18017/) | Hereditary antithrombin deficiency | no assertion criteria provided |
| [c.1306G>A (p.Ala436Thr)](https://www.ncbi.nlm.nih.gov/clinvar/variation/18003/) | Hereditary antithrombin deficiency | Pathogenic(Aug 9, 1988) |
| [c.1274G>A (p.Arg425His)](https://www.ncbi.nlm.nih.gov/clinvar/variation/18019/) | Hereditary antithrombin deficiency | Pathogenic(Oct 29, 2018) |
| [c.1274G>C (p.Arg425Pro)](https://www.ncbi.nlm.nih.gov/clinvar/variation/18009/) | Hereditary antithrombin deficiency | Pathogenic(Jan 28, 2020) |
| [c.1273C>T (p.Arg425Cys)](https://www.ncbi.nlm.nih.gov/clinvar/variation/18016/) | Hereditary antithrombin deficiency | Pathogenic(Jan 1, 1989) |
| [c.1271G>A (p.Gly424Asp)](https://www.ncbi.nlm.nih.gov/clinvar/variation/18033/) | Hereditary antithrombin deficiency | Pathogenic(Feb 24, 2019) |
| [c.1246G>C (p.Ala416Pro)](https://www.ncbi.nlm.nih.gov/clinvar/variation/18007/) | Hereditary antithrombin deficiency | Pathogenic(Nov 1, 1992) |
| [c.1240G>A (p.Ala414Thr)](https://www.ncbi.nlm.nih.gov/clinvar/variation/18020/) | Hereditary antithrombin deficiency | Pathogenic(Aug 15, 1990) |
| [c.1219-2A>G](https://www.ncbi.nlm.nih.gov/clinvar/variation/1527905/) | Hereditary antithrombin deficiency | Pathogenic(Jan 1, 1996) |
| [c.1190C>G (p.Ser397Ter)](https://www.ncbi.nlm.nih.gov/clinvar/variation/1068471/) | Hereditary antithrombin deficiency | Pathogenic(Feb 11, 2022) |
| [c.1157T>C (p.Ile386Thr)](https://www.ncbi.nlm.nih.gov/clinvar/variation/863657/) | Hereditary antithrombin deficiency | Pathogenic(Oct 15, 2020) |
| [c.1154-14G>A](https://www.ncbi.nlm.nih.gov/clinvar/variation/410384/) | Hereditary antithrombin deficiency | Pathogenic (Aug 23, 2021) |
| [c.1141T>C (p.Ser381Pro)](https://www.ncbi.nlm.nih.gov/clinvar/variation/18032/) | Hereditary antithrombin deficiency | Pathogenic (Mar 3, 2020) |
| [c.1016G>A (p.Trp339Ter)](https://www.ncbi.nlm.nih.gov/clinvar/variation/100923/) | Hereditary antithrombin deficiency | Pathogenic(Mar 1, 1992) |
| [c.685C>T (p.Arg229Ter)](https://www.ncbi.nlm.nih.gov/clinvar/variation/1455332/) | Hereditary antithrombin deficiency | Pathogenic(Jul 7, 2021) |
| [c.667T>C (p.Ser223Pro)](https://www.ncbi.nlm.nih.gov/clinvar/variation/18043/) | Hereditary antithrombin deficiency | Pathogenic(Jul 18, 2021) |
| [c.655A>G (p.Asn219Asp)](https://www.ncbi.nlm.nih.gov/clinvar/variation/18042/) | Hereditary antithrombin deficiency | Pathogenic(Nov 1, 2001) |
| [c.624+1G>A](https://www.ncbi.nlm.nih.gov/clinvar/variation/694627/) | Hereditary antithrombin deficiency | Pathogenic (Jun 10, 2020) |
| [c.607C>T (p.Gln203Ter)](https://www.ncbi.nlm.nih.gov/clinvar/variation/1456446/) | Hereditary antithrombin deficiency | Pathogenic(Nov 8, 2019) |
| [c.500A>C (p.Asn167Thr)](https://www.ncbi.nlm.nih.gov/clinvar/variation/18041/) | Hereditary antithrombin deficiency | Pathogenic(Oct 14, 2021) |
| [c.481C>T (p.Arg161Ter)](https://www.ncbi.nlm.nih.gov/clinvar/variation/18029/) | Hereditary antithrombin deficiency | Pathogenic(Jun 15, 1999) |
| [c.442T>C (p.Ser148Pro)](https://www.ncbi.nlm.nih.gov/clinvar/variation/18039/) | Hereditary antithrombin deficiency | Pathogenic(Aug 23, 2019) |
| [c.436A>G (p.Lys146Glu)](https://www.ncbi.nlm.nih.gov/clinvar/variation/1330290/) | Hereditary antithrombin deficiency | Pathogenic(Mar 1, 1993) |
| [c.391C>T (p.Leu131Phe)](https://www.ncbi.nlm.nih.gov/clinvar/variation/18034/) | Hereditary antithrombin deficiency, Deep venous thrombosis | Pathogenic(Dec 3, 2021) |
| [c.381T>A (p.Cys127Ter)](https://www.ncbi.nlm.nih.gov/clinvar/variation/1434089/) | Hereditary antithrombin deficiency | Pathogenic(Feb 14, 2022) |
| [c.379T>C (p.Cys127Arg)](https://www.ncbi.nlm.nih.gov/clinvar/variation/18044/) | Hereditary antithrombin deficiency | Pathogenic(Sep 1, 2021) |
| [c.341G>A (p.Ser114Asn)](https://www.ncbi.nlm.nih.gov/clinvar/variation/843350/) | Hereditary antithrombin deficiency | Pathogenic(Dec 27, 2002) |
| [c.235C>A (p.Arg79Ser)](https://www.ncbi.nlm.nih.gov/clinvar/variation/18015/) | Hereditary antithrombin deficiency | Pathogenic(Nov 28, 2019) |
| [c.235C>T (p.Arg79Cys)](https://www.ncbi.nlm.nih.gov/clinvar/variation/18004/) | Hereditary antithrombin deficiency | Pathogenic(Jun 18, 1990) |
| [c.218C>T (p.Pro73Leu)](https://www.ncbi.nlm.nih.gov/clinvar/variation/18011/) | Hereditary antithrombin deficiency | Pathogenic(Jan 3, 2022) |
| [c.159C>A (p.Cys53Ter)](https://www.ncbi.nlm.nih.gov/clinvar/variation/627247/) | Deep venous thrombosis | Pathogenic(Dec 8, 2021) |
| [c.116T>A (p.Ile39Asn)](https://www.ncbi.nlm.nih.gov/clinvar/variation/18021/) | Hereditary antithrombin deficiency | Pathogenic(Feb 1, 2019) |
| [c.68T>C (p.Leu23Pro)](https://www.ncbi.nlm.nih.gov/clinvar/variation/18040/) | Hereditary antithrombin deficiency | Pathogenic(Jan 1, 1996) |
